# Supplementary material for: Phase I/II trial of a peptide-based COVID-19 T-cell activator in patients with B-cell deficiency
Source: Nat Commun. 2023 Aug 18;14:5032. doi: 10.1038/s41467-023-40758-0 (PMC10439231; doi:10.1038/s41467-023-40758-0)
Supplement: Supplementary file 1 — Supplementary Information [file 41467_2023_40758_MOESM1_ESM.pdf]

Supplementary Information to the Manuscript Entitled

Phase I/II trial of a peptide-based COVID-19 T-cell activator in patients with B-cell deficiency

Table of Contents

Supplementary Methods 2

Detailed inclusion and exclusion criteria for trial participants 2
Statistical considerations including sample size and holding rules 3
Immunocompetent SARS-CoV-2 convalescent individuals and B-cell deficient mRNA vaccinated patients 5
IFN-γ ELISPOT assay *ex vivo* or following 12-day *in vitro* expansion 5
Intracellular cytokine and cell surface marker staining 6
Antibody testing 7
Software and statistical analysis 8
Design of CoVac-1 8

Supplementary Notes 9

Supplementary Note 1: Safety laboratory assessment 9
Supplementary Note 2: Unsolicited adverse events 9
Supplementary Note 3: Outcome of SARS-CoV-2 positivity during trial 10
Supplementary Note 4: Interim assessment of Phase I 11

Supplementary Tables 12

Supplementary Table 1: Toxicity grading scale modified according to CTCAE V5.0 12
Supplementary Table 2: Potential immune mediated medical conditions 13
Supplementary Table 3: Adverse events of special interest (AESI) 14
Supplementary Table 4: Unsolicited AEs, AESIs, and SAEs until day 56, classified according to CTCAE V5.0. 15
Supplementary Table 5: HLA-DR viral control peptide panel (ADV, CMV, EBV) 18
Supplementary Table 6: Characteristics of immunocompetent convalescents after SARS-CoV-2 infection 19
Supplementary Table 7: Additional information on patients` prior vaccination 20
Supplementary Table 8: Hematologic malignancies and therapies of patients included in the study 21
Supplementary Table 9: Mutations of SARS-CoV-2 omicron variants 22

Supplementary Figures 23

Supplementary Figure 1: Intensities of CoVac-1-induced T-cell responses *ex vivo* assessed in IFN-γ ELISPOT assays. 23
Supplementary Figure 2: Intensities of CoVac-1-induced T-cell responses assessed in IFN-γ ELISPOT assays after 12-day *in vitro* expansion 25
Supplementary Figure 3: CoVac-1-induced $CD4^+$ T-cell responses in study participants 27
Supplementary Figure 4: Gating strategy for flow cytometry-based evaluation of surface marker and intracellular cytokine staining 28

Supplementary references 29

Supplementary Methods

Detailed inclusion and exclusion criteria for trial participants

Eligible participants were men or women aged 18 years or above, who signed the informed consent form. In addition, patients had primary or secondary antibody deficiency syndrome, defined by (i) IgG < 5.5 g/l, (ii) ongoing substitution of immunoglobulins for hypogammaglobulinemia, (iii) ongoing or up to 6 months after single agent anti-CD20 antibody therapy, (iv) ongoing or up to 6 months after combined anti-CD20 antibody therapy with Bruton`s tyrosine kinase (BTK)-inhibitors or B-cell lymphoma 2 (BCL2)-inhibitors, (v) after combined anti-CD20 antibody therapy with chemotherapy (e.g. fludarabine, cyclophosphamide, bendamustine, anthracycline, vincristine) (within 1-6 month post therapy) or (vi) anti-CD20 antibody maintenance therapy. All participants had to adhere to adequate contraception methods until three months after vaccination.

Exclusion criteria comprised: Pregnant or lactating females; participation in another clinical trial with a vaccine product; prior or current infection with SARS-CoV-2 (proven serologically or by PCR); persisting symptoms developed after vaccination against SARS-CoV-2 with one of the approved vaccines products; intention of receiving one dose of an already approved vaccine against SARS-CoV-2 before day 56; known previous anaphylactic reaction or hypersensitivity to any component of CoVac-1; patients presenting clinical, laboratory or radiological signs of tumor-progression; patients receiving active treatment with proteosome-inhibitors (e.g. bortezomib), or phosphoinositide-3-kinase-inhibors (e.g. idelalisib); relevant central nervous system (CNS) pathology or other neurological disease; positivity for human immunodeficieny virus (HIV) or active hepatitis; baseline $CD4^+$ T-cell count ≤ 100/µL; chronic liver failure defined as Child-Pugh Score ≥ B; chronic renal failure (GFR < 40 mL/min/1.73 m^2); serious cardiovascular disease (NYHA ≥ III); sickle cell anemia; or pre-existing auto-immune disease except for Hashimoto thyroiditis and mild psoriasis.

Statistical considerations including sample size and holding rules

The total sample size calculation (n = 54 patients) of the trial was based on the following assumptions: The Phase I of the study will comprise 14 - 28 patients depending on the interim analysis. In Phase I of the study the safety/toxicity of CoVac-1 in patients with B-cell/antibody deficiency will be investigated. For this purpose, it will be investigated whether the incidence of severe adverse events (SAE) associated with administration of CoVac-1 exceeds a predetermined rate of 20% (= P1 = alternative hypothesis) in the study population (14 patients). Safety of CoVac-1 is shown if no (Phase I) CoVac-1-related SAE (= P0 = null hypothesis) occurs in the study population. The safety and toxicity of CoVac-1 will be determined based on the Common Terminology Criteria for Adverse Events (CTCAE V5.0) and assessed in a descriptive manner. If there is an insufficient T-cell response (< 80% of patients, with the general ability to mount antigen-specific T cell responses, show CoVac-1-specific T-cell responses) measured by IFN-γ ELISPOT in Phase I on day 28, an additional part of Phase I will start enrollment of additional 14 subjects receiving two injections of CoVac-1 on day 1 and day 42.

The sample size of the Phase II part of the study was chosen based on the assumption that, in the unfavorable case of SARS-CoV-2-specific immune response induction in ≤ 50% (P0) of the patients, the vaccine concept is extended with a probability of at most 5% (type one error, one-sided). On the other hand, in the favorable case of peptide-specific immune response induction in ≥ 70% (P1) of the patients, the therapy concept should be followed with a probability of at least 80% (power). A sample size of 37 achieves 80.51% power to detect a difference (P1-P0) of 0.2000 using a one-sided exact test with a significance level (alpha) of 0.05. These results assume that the population proportion under the null hypothesis (P0) is 0.50.

In this clinical trial the following predefined holding rules were implemented:

- Solicited local adverse drug reactions (ADRs): If more than 30% of injections are followed by grade ≥ 3 solicited swelling or pain or grade 4 redness occurring for the first time after injection (day of injection until day 28) and persisting at grade 3 (swelling or pain) / 4 (redness) for > 48 hours to maximum 72 hours depending upon symptom severity and kinetics.
- Solicited systemic adverse events (AEs): If more than 25% of injections are followed by grade 3 solicited systemic AE beginning within 3 days after study injection (day of injection and 2 subsequent days) and persisting at grade ≥ 3 for > 48 hours to maximum 72 hours depending upon symptom severity and kinetics.
- Unsolicited AEs: If more than 25% of patients develop a grade ≥ 3 unsolicited AE (including laboratory AE and physical observations) that is considered probably or definitely related to injection and persists at grade 3 for > 48 hours to maximum 72 hours depending upon symptom severity and kinetics.
- SAE (including suspected unexpected serious adverse drug reaction, SUSAR) that is life-threatening or results in death.

If a holding rule is met, further recruitment is paused until review by the data safety monitoring board (DSMB). In addition, the DSMB reviewed the safety and immunogenicity data of the Phase I of the clinical trial prior to proceeding to Phase II.

Immunocompetent SARS-CoV-2 convalescent individuals and B-cell deficient mRNA vaccinated patients

To compare CoVac-1-induced T-cell responses in study patients to SARS-CoV-2-specific T-cell responses after natural infection, a reference group of non-hospitalized COVID-19 convalescent individuals (healthy COVID-19 convalescents (HCs))[1-3], was used. SARS-CoV-2 infection was confirmed by real-time polymerase chain reaction (PCR) in nasopharyngeal swabs. Sample collection for (n = 61) was performed 16-66 days after positive PCR. Written informed consent was obtained in accordance with the Declaration of Helsinki (local Ethics Committee at University Hospital Tübingen; project number: 179/2020/BO2). Details are provided in Table S6.

In addition, CoVac-1-induced T-cell responses were compared to spike-specific T-cell responses induced by approved mRNA-based vaccines in B-cell deficient patients prior to CoVac-1 application. Sample collection was performed 66 days (median) after the last vaccination. Details are provided in Table S7.

Peripheral blood mononuclear cells (PBMCs) were isolated by density gradient centrifugation and were stored at -80°C.

IFN-γ ELISPOT assay *ex vivo* or following 12-day *in vitro* expansion

Peptide-stimulated (*in vitro* expanded) or freshly thawed (*ex vivo*) PBMCs were analyzed by interferon (IFN)-γ enzyme-linked immunospot (ELISPOT) assay. 1.5 - 5 x 10^5 cells per well (technical replicates) were incubated in 96-well ELISPOT plates coated with anti-IFN-γ antibody (clone 1-D1K, 2 µg/mL, MabTech, Cat# 3420-3-250, RRID: AB_907283) with 2.5 µg/mL of CoVac-1 peptides and 1 µg/mL for overlapping 15-mer peptide mix covering the entire spike protein (Milteny PepTivator® SARS-CoV-2 Prot_S, PepTivator® SARS-CoV-2 Prot_S+, PepTivator® SARS-CoV-2 Prot_S1), respectively. PHA (Sigma-Aldrich) served as positive control. An irrelevant HLA-DR-restricted control peptide (ETVITVDTKAAGKGK,

FLNA_HUMAN$_{1669–1683}$) or 10% dimethyl sulfoxide (DMSO) in double-distilled water served as negative controls. After 24 hours of incubation, spots were revealed with anti-IFN-γ biotinylated detection antibody (clone 7-B6-1, 0.3 µg/mL, MabTech, Cat# 3420-6-250, RRID: AB_907273), ExtrAvidin-Alkaline Phosphatase (1:1,000 dilution, Sigma-Aldrich), and BCIP/NBT (5-bromo-4-chloro-3-indolyl-phosphate/nitro-blue tetrazolium chloride, Sigma-Aldrich). Spots were counted using an ImmunoSpot S6 analyzer (CTL). Patients without the general ability to mount antigen-specific T-cell responses (absence of CoVac-1-induced T-cell responses and no T-cell responses to HLA-DR T-cell epitope control panel including Epstein-Barr virus (EBV), cytomegalovirus (CMV) and adenovirus (ADV) peptides[4], Table S5) were considered as not assessable (drop-out). T-cell responses were considered positive (indicated as median of positive samples (pos)) if the mean spot count of the technical replicates was ≥ 3-fold higher than the mean spot count of the negative control and defined as CoVac-1-induced (indicated as response (%)) if the mean spot count post vaccination was ≥ 2-fold higher than the respective spot count on day 1. If not otherwise indicated, the intensity of T-cell responses is depicted as calculated spot counts, which represent the mean spot count minus the mean spot count of the respective negative control, normalized to the indicated amount of cells.

Intracellular cytokine and cell surface marker staining

Peptide-specific T cells were characterized by cell surface marker and intracellular cytokine staining (ICS). PBMCs were incubated with the CoVac-1 vaccine peptide pool or the negative control peptide (10 µg/mL per peptide), Brefeldin A (Sigma-Aldrich), and GolgiStop (BD Biosciences). PMA and ionomycin (Sigma-Aldrich) served as positive control. Staining was performed using Cytofix/Cytoperm solution (BD), Aqua live/dead (1:400 dilution, Invitrogen), APC/Cy7 anti-human CD4 (1:100 dilution, BioLegend, Cat# 300518, RRID: AB_314086), PE/Cy7 anti-human CD8 (1:400 dilution, Beckman Coulter, Cat# 737661, RRID: AB_1575980), Pacific Blue anti-human tumor necrosis factor (TNF, 1:120 dilution,

BioLegend, Cat# 502920, RRID: AB_528965), FITC anti-human CD107a (1:100 dilution, BioLegend, Cat# 328606, RRID: AB_1186036), APC anti-human IL-2 (1:40 dilution, BioLegend, Cat# 500309, RRID: AB_315096), and PE anti-human IFN-γ monoclonal antibodies (1:200 dilution, BioLegend, Cat# 506507, RRID: AB_315440). T-cell responses were considered positive (indicated as median of positive samples (pos)) if the detected frequency of cytokine-positive $CD4^+$ T cells was ≥ 3-fold higher than the frequency in the negative control. Frequency of cytokine-positive cells was corrected for background by subtraction of the respective negative control values. Negative values were set to zero. Results were defined as induced response (depicted as response (%)) if the frequency of cytokine-positive cells was ≥ 2-fold higher than the respective frequency on day 1. All samples were analyzed on a FACS Canto II cytometer (BD). The gating strategy applied for the analyses of flow cytometry-acquired data is provided in Fig. S4.

Antibody testing

The Siemens SARS-CoV-2 IgG (SCOVG) assay was performed on an automated ADVIA Centaur XPT system (Siemens Healthineers) according to the manufacturer's instructions. The immunoassays detect anti-SARS-CoV-2 IgG antibodies (SCOVG) directed against the S1 domain of the viral spike protein (including the immunologically relevant receptor binding domain). Results of one measurement of each serum sample are reported in Index Values. The final interpretation of positivity is determined by an Index Value ≥ 1.0 given by the manufacturer. Values < 0.1 were set to 0.1. Quality control was performed following the manufacturer's instructions on each day of testing.

Software and statistical analysis

Flow cytometric data was analyzed using FlowJo 10.7.1 (BD). Graphs were plotted using Inkscape 1.1 and GraphPad Prism 9.2.0. Statistical analyses were conducted using GraphPad Prism 9.2.0 and SAS Version 9.4. CoVac-1 peptide-binding prediction was performed using the prediction algorithm NetMHCIIpan 4.2a[5] with all available allele combinations (n = 2967 for HLA-DR, n = 13233 for HLA-DQ, n = 1562 for HLA-DP) and the threshold of percentile rank < 5.

Design of CoVac-1

CoVac-1 is a peptide-based T-cell activator comprising six HLA-DR-restricted SARS-CoV-2 peptides[3] predicted to bind to the common HLA-DR alleles HLA-DRB1*01, -DRB1*04, -DRB1*07, -DRB1*11, -DRB1*15 and derived from various SARS-CoV-2 proteins (spike, nucleocapsid, membrane, envelope, and ORF8). CoVac-1 peptides are adjuvanted with the novel toll-like receptor (TLR) 1/2 agonist XS15 emulsified in Montanide™ ISA51 VG, which endorse activation and maturation of antigen presenting cells and prevent vaccine peptides from immediate degradation, enabling the induction of a potent T-cell response[6-8]. CoVac-1 HLA-DR T-cell epitopes contain embedded HLA class I sequences for induction of both, $CD4^+$ and $CD8^+$ T-cell responses. CoVac-1 peptides were selected from viral non-surface proteins and their subunits or - in case of the spike protein-derived T-cell epitope P3_spi - from buried/hidden amino acid sequences, which are not accessible for antibodies in their conformational state. The linear 15-amino acid peptides are characterized by a free N-terminal amino group and a free C-terminal carboxy group. All amino acid residues are in the L-configuration and not chemically modified at any position. Synthetic peptides were manufactured by established solid phase peptide synthesis procedures using Fmoc chemistry[9,10].

Supplementary Notes

Supplementary Note 1: Safety laboratory assessment

Safety laboratory AEs were graded according to a CTCAE V5.0 grading scale. Abnormal laboratory parameters were assessed by the investigators for clinical significance. Chemotherapy-induced grade 4 neutropenia was observed in one patient and assessed not related to CoVac-1. No other clinically significant laboratory deviation was reported. If normal at baseline, deviations from normal range were reported for eight parameters (hemoglobulin, creatinine, bilirubin, alkaline phosphatase, alanine transaminase, aspartate transaminase, lactate dehydrogenase, and C-reactive protein). The most frequent deviation was reported for C-reactive protein in 9 participants (17%). 3 patients were reported to have grade 1 elevated liver enzymes.

Supplementary Note 2: Unsolicited adverse events

In case an AE, even if determined by definition as solicited (local solicited: erythema, swelling, itching, pain, induration/granuloma, lymphadenopathy, skin ulceration; systemic solicited: e.g. arthralgia, chills, fatigue, fever, gastrointestinal symptoms, headache, myalgia), was clearly caused by another reason, the investigator judged the event as unrelated to CoVac-1. These unrelated AEs are displayed as unsolicited AEs (Table S4).

There were 67 unsolicited AEs reported, of which three were reported as SAE. Of all unsolicited AEs, 79% were mild in severity, 3 of which were ruled as related to vaccination (shingles, herpes simplex reactivation and blister at administration site), and 12% and 7% were moderate and severe, respectively, of which none was judged as related to vaccination; and 1% was life-threatening (chemotherapy-induced grade 4 neutropenia), which was not related to vaccination. The three SAEs comprised one COVID-19 infection (as described in detail above), surgical removal of the prostate (due to suspicious lesions on imaging) revealing histological confirmed

prostate cancer and a fracture of the shoulder after a bicycle accident, which was surgically treated.

Supplementary Note 3: Outcome of SARS-CoV-2 positivity during trial

Until day 56 two subjects were infected with SARS-CoV-2 and experienced a mild course of COVID-19. The first patient suffered from chronic lymphocytic leukemia and received treatment with the BKT-inhibitor acalabrutinib. The patient was included due to low serum IgG levels (< 5.5g/l) at the Frankfurt study site. The patient was diagnosed with COVID-19 14 days after CoVac-1 application, SARS-CoV-2 variant analysis was not available. Symptoms were mild and included fever (38.4°C) for two days and chest pain for three days, which resolved without sequel. No COVID-19 specific treatment was initiated, symptomatic treatment with ibuprofen was applied once. The patient recovered from symptoms within four days after onset of symptoms.

The second patient suffered from diffuse large B-cell lymphoma and received treatment with immunochemotherapy, which had been finished about 1 month prior to CoVac-1 administration. The patient was included due to combined anti-CD20 antibody therapy with chemotherapy (within 1-6 months post therapy) at the Tübingen study site. Patient experienced symptoms starting 40 days after CoVac-1 application. Symptoms were mild and included fever (up to 39.4°C) for two days, sore throat, headache and mild cough. No dyspnea was reported. Two days after onset of symptoms PCR test was performed, which revealed infection with the omicron variant (carrying the mutation Y505H). A chest computed tomography (CT) scan did not show any signs of severe pneumonia; laboratory assessment showed no signs of inflammation. Based on individual risk assessment of the patient (time from last chemoimmunotherapy to infection), the treating hematologist decided to apply sotrovimab 500 mg on day five after onset of symptoms (hospitalization for one night). At that time symptoms were still mild (cough and sore throat), and the fever had already resolved. The AESI was terminated 14 days after onset of symptoms.

Supplementary Note 4: Interim assessment of Phase I

A single-center run-in Phase I part of the trial was planned to assess for safety and efficacy in terms of induction of T-cell responses to CoVac-1 in the trial population of B-cell deficient patients. The safety assessment was performed on day 28 after CoVac-1 application. Until day 28 no relevant inflammatory systemic side effects, especially no fever was reported. No allergic reactions were observed. As intended and expected 13 volunteers (93%) developed a granuloma at the injection site (max. grade 2). Further local injection site adverse events were mild to moderate and included transient erythema (86%), swelling (43%), itching (29%), pain (50%). Vaccination site lymphadenopathy was observed in 14% of study subjects. Immunogenicity, in term of induction of T-cell responses to one or more of the six HLA-DR SARS-CoV-2 T-cell epitopes included in the CoVac-1 was assessed at baseline as well as on day 7, 14 and 28 after CoVac-1 application for all subjects in Phase I of the study. Induction of SARS-CoV-2-specific T cells was shown in 93% (13/14) of study patients on day 28 analyzed by *ex vivo* IFN-γ ELISPOT assay. Thus, high immunogenicity of CoVac-1 to induce early and multi-peptide-specific T-cell responses in patients with congenital or acquired B-cell deficiency was shown. After the interim analysis and review by the DSMB, it was decided to proceed to Phase II without further modifying of the dose schedule. The amendment was approved by the regulatory authorities and the local Ethics Committees.

Supplementary Tables

Supplementary Table 1: Toxicity grading scale modified according to CTCAE V5.0.

Local solicited AEs	CTCAE Term	Grade 0 (normal)	Grade 1 (mild)	Grade 2 (moderate)	Grade 3 (severe)	Grade 4 (life-threatening)
**Erythema**	Vaccination complication	< 25 mm	25 - 50 mm<br>Tenderness with or without associated symptoms (e.g., warmth, itching)	51 - 100 mm<br>Pain; lipodystrophy; edema; phlebitis	> 100 mm or operative intervention indicated	Life-threatening consequences: urgent intervention indicated
**Swelling**	Vaccination complication	< 25 mm	25 - 50 mm and does not interfere with activity	> 50 mm	Prevents daily activity	Necrosis
**Pain**	Vaccination complication	None	Tenderness with or without associated symptoms<br><br>Does not interfere with activity	Pain; lipodystrophy; edema; phlebitis<br><br>Interferes with activity	Prevents daily activity	Life-threatening consequences: urgent intervention indicated emergency room visit or hospitalization
**Induration/ granuloma**	Vaccination complication	None	< 50 mm	51 - 100 mm	> 100 mm or operative intervention indicated	Life-threatening consequences: urgent intervention indicated
**Ulceration**	Vaccination complication	None	Combined area of ulcers < 1 cm	Combined area of ulcers 1 - 2 cm	Combined area of ulcers > 2 cm or operative intervention indicated	Necrosis

Abbreviations: AE, adverse event; CTCAE, Common Terminology Criteria for Adverse Events.

Supplementary Table 2: Potential immune-mediated medical conditions[11].

Neuroinflammatory disorders	Musculoskeletal disorders	Skin disorders	Liver disorder	Gastrointestinal disorders	Metabolic & endocrine disorders	Vasculitides	Others
Cranial nerve inflammatory disorders, including paralyses/paresis (e.g. Bell's palsy)	Systemic lupus erythematosus	Psoriasis	Autoimmune hepatitis	Crohn's disease	Autoimmune thyroiditis including Hashimoto thyroiditis	Large vessels vasculitis, including giant cell arteritis such as Takayasu's arteritis and temporal arteritis	Autoimmune haemolytic anaemia
Acute disseminated encephalomyelitis, including site-specific variants: encephalitis, encephalomyelitis, myelitis, myeloradiculoneuritis, cerebellitis	Systemic sclerosis (with limited or diffuse cutaneous involvement)	Vitiligo	Primary biliary cirrhosis	Ulcerative colitis	Grave's or Basedow's disease		Autoimmune thrombocytopenia
Multiple sclerosis	Dermatomyositis	Erythema nodosum	Primary sclerosing cholangitis	Ulcerative proctitis	Diabetes mellitus type I		Antiphospholipid syndrome
Transverse myelitis	Polymyositis		Autoimmune cholangitis	Celiac disease	Addison's disease		Pernicious anaemia
Optic neuritis	Anti-synthetase syndrome	Cutaneous lupus erythematosus					Raynaud's phenomenon
Narcolepsy	Rheumatoid arthritis	Alopecia areata					Uveitis
	Juvenile chronic arthritis including Still's disease	Lichen planus					Autoimmune myocarditis/cardiomyopathy
	Polymyalgia rheumatica	Sweet's syndrome					Sarcoidosis
	Psoriatic arthropathy	Morphoea					Stevens-Johnson syndrome
	Relapsing polychondritis						Sjögren's syndrome
Myasthenia gravis including Lambert-Eaton myasthenic syndrome	Mixed connective tissue disorder						Idiopathic pulmonary fibrosis
							Goodpasture syndrome
Immune-mediated peripheral neuropathies and plexopathies including Guillain-Barré syndrome, Miller Fisher syndrome and other variants, chronic inflammatory demyelinating polyneuropathy, multifocal motor neuropathy and polyneuropathies associated with monoclonal gammopathy	Spondyloarthritis including ankylosing spondylitis, reactive arthritis (Reiter's Syndrome) and undifferentiated spondyloarthritis	Autoimmune bullous skin diseases including pemphigus, pemphigoid and dermatitis herpetiformis				Medium sized and/or small vessels vasculitis including polyarteritis nodosa, Kawasaki's disease, microscopic polyangiitis, Wegener's granulomatosis, Churg–Strauss syndrome (allergic granulomatous angiitis), Buerger's disease (thromboangiitis obliterans), necrotising vasculitis and anti-neutrophil cytoplasmic antibody (ANCA) positive vasculitis (type unspecified), Henoch-Schonlein purpura, Behcet's syndrome, leukocytoclastic vasculitis	Autoimmune glomerulonephritis including IgA nephropathy, glomerulonephritis rapidly progressive, membranous glomerulonephritis, membranoproliferative glomerulonephritis, and mesangioproliferative glomerulonephritis

Supplementary Table 3: Adverse events of special interest (AESI).

AESI term
Novel proven (PCR-based) SARS-CoV-2 infection accompanied by symptoms
Novel proven (PCR-based) SARS-CoV-2 positivity without symptoms
Novel potential immune-mediated condition

Abbreviation: PCR, polymerase chain reaction.

Supplementary Table 4: Unsolicited AEs, AESIs, and SAEs until day 56, classified according to CTCAE V5.0.

CTCAE	Severity	All participants (n = 54)		Phase I (n = 14)		Phase II (n = 40)	
		Not related to vaccine	Related to vaccine	Not related to vaccine	Related to vaccine	Not related to vaccine	Related to vaccine
Any event	Mild	50	3	14	-	36	3
	Moderate	8	-	3	-	5	-
	Severe	5	-	1	-	4	-
	Life-threatening	1	-	-	-	1	-
Arthralgia	Mild	1	-	-	-	1	-
	Moderate	-	-	-	-	-	-
Atrial fibrillation	Mild	1	-	-	-	1	-
	Moderate	-	-	-	-	-	-
Blister	Mild	-	1	-	-	-	1
	Moderate	-	-	-	-	-	-
Bloating	Mild	1	-	1	-	-	-
	Moderate	-	-	-	-	-	-
Bone pain	Mild	1	-	-	-	1	-
	Moderate	-	-	-	-	-	-
Chills	Mild	1	-	1	-	-	-
	Moderate	-	-	-	-	-	-
Chough	Mild	1	-	-	-	1	-
	Moderate	1	-	-	-	1	-
Diarrhea	Mild	2	-	1	-	1	-
	Moderate	-	-	-	-	-	-
Dizziness	Mild	2	-	1	-	1	-
	Moderate	1	-	-	-	1	-
Dyspnea	Mild	2	-	1	-	1	-
	Moderate	-	-	-	-	-	-
Ear pain	Mild	1	-	-	-	1	-
	Moderate	-	-	-	-	-	-
Edema limbs	Mild	1	-	-	-	1	-
	Moderate	-	-	-	-	-	-
Edema of both legs	Mild	1	-	-	-	1	-
	Moderate	-	-	-	-	-	-
Epistaxis	Mild	1	-	-	-	1	-
	Moderate	-	-	-	-	-	-
Fatigue	Mild	1	-	1	-	-	-
	Moderate	-	-	-	-	-	-
Flu like symptoms	Mild	4	-	1	-	3	-
	Moderate	-	-	-	-	-	-
Fracture	Mild	-	-	-	-	-	-
	Moderate	-	-	-	-	-	-
	Severe	1	-	1	-	-	-
Headache	Mild	2	-	1	-	1	-
	Moderate	-	-	-	-	-	-

CTCAE	Severity	All participants (n = 54)		Phase I (n = 14)		Phase II (n = 40)	
		**Not related to vaccine**	**Related to vaccine**	**Not related to vaccine**	**Related to vaccine**	**Not related to vaccine**	**Related to vaccine**
Hearing impaired	Mild	-	-	-	-	-	-
	Moderate	1	-	1	-	-	-
Herpes simplex reactivation	Mild	-	1	-	-	-	1
	Moderate	-	-	-	-	-	-
Hot flashes	Mild	2	-	-	-	2	-
	Moderate	-	-	-	-	-	-
Hypertension	Mild	-	-	-	-	-	-
	Moderate	1	-	1	-	-	-
	Severe	1	-	-	-	1	-
Infections and infestations, other - novel proven SARS-CoV-2 infection	Mild	2*, **	-	-	-	2*, **	-
	Moderate	-	-	-	-	-	-
Insomnia	Mild	1	-	-	-	1	-
	Moderate	-	-	-	-	-	-
Lyme borreliosis	Mild	1	-	1	-	-	-
	Moderate	-	-	-	-	-	-
Lymphadenopathy	Mild	1	-	1	-	-	-
	Moderate	-	-	-	-	-	-
Menorrhagia	Mild	-	-	-	-	-	-
	Moderate	1	-	-	-	1	-
Muscle cramp	Mild	1	-	-	-	1	-
	Moderate	-	-	-	-	-	-
Nausea	Mild	6	-	1	-	5	-
	Moderate	-	-	-	-	-	-
Neoplasms benign, malignant and unspecified (incl. cysts and polyps) - others, prostate	Mild	-	-	-	-	-	-
	Moderate	-	-	-	-	-	-
	Severe	1*	-	-	-	1*	-
Neutrophile count decreased	Mild	-	-	-	-	-	-
	Moderate	-	-	-	-	-	-
	Severe	-	-	-	-	-	-
	Life-threatening	1	-	-	-	1	-
Pain in extremity – left knee	Mild	1	-	-	-	1	-
	Moderate	-	-	-	-	-	-
Pain in extremity – right upper arm	Mild	1	-	-	-	1	-
	Moderate	-	-	-	-	-	-
Periodontal disease	Mild	1	-	-	-	1	-
	Moderate	-	-	-	-	-	-
Presyncope	Mild	-	-	-	-	-	-
	Moderate	1	-	1	-	-	-
Renal calculi	Mild	-	-	-	-	-	-
	Moderate	-	-	-	-	-	-
	Severe	1	-	-	-	1	-
Shingles	Mild	-	1	-	-	-	1
	Moderate	-	-	-	-	-	-
Sinusitis	Mild	1	-	1	-	-	-
	Moderate	1	-	-	-	1	-

CTCAE	Severity	All participants (n = 54)		Phase I (n = 14)		Phase II (n = 40)	
		Not related to vaccine	Related to vaccine	Not related to vaccine	Related to vaccine	Not related to vaccine	Related to vaccine
Sore throat	Mild	2	-	-	-	2	-
	Moderate	-	-	-	-	-	-
Stomach pain	Mild	2	-	1	-	1	-
	Moderate	-	-	-	-	-	-
Suspicious skin lesion	Mild	-	-	-	-	-	-
	Moderate	1	-	-	-	1	-
Vaginal infection	Mild	1	-	-	-	1	-
	Moderate	-	-	-	-	-	-
Vasovagal reaction	Mild	-	-	-	-	-	-
	Moderate	-	-	-	-	-	-
	Severe	1	-	-	-	1	-
Vertigo	Mild	2	-	1	-	1	-
	Moderate	-	-	-	-	-	-
Vomiting	Mild	2	-	-	-	2	-
	Moderate	-	-	-	-	-	-

Severity and relationship were judged by the investigator until day 56. *termed as SAE, **termed as AESI. Abbreviations: AE, adverse event; AESI, adverse event of special interest; SAE, serious adverse event; CTCAE, common terminology criteria for adverse events; n, number.

Supplementary Table 5: HLA-DR viral control peptide panel (ADV, CMV, EBV).

Peptide sequence	Source protein	Organism
AQYIKANSKFIGITEL	TETX	CLOTE
RSPTVFYNIPPMPLPPSQL	EBNA2	EBV
PRPVSRFLGNNSILY	GP350	EBV
VPYIKWDNCNSTNIT	BLLF1	EBV
PIFIRRLHRLLLMRA	EBNA3	EBV
TFYLNHTFKK	HEX	ADV
GTAYNALAPKGAPNP	HEX	ADV
HPTFTSQYRIQGKLEYR	pp65	CMV
YQEFFWDANDIYRIF	pp65	CMV
STNITAVVRAQGLDV	GP350	EBV
KTSLYNLRRGTALA	EBN1	EBV
RGIFCVVKQAKLTYE	E3145	ADV
VSKFFHAFPSKLHDK	PKG1	ADV

Abbreviations: ADV, adenovirus; CMV, cytomegalovirus; EBV, Epstein-Barr virus; CLOTE, *Clostridium tetani*.

Supplementary Table 6: Characteristics of immunocompetent convalescents after SARS-CoV-2 infection[2].

Characteristics	HCs with humoral immune response after infection		
	CoVac-1	SARS-CoV-2-specific EC	Cross-reactive EC
Samples – no.	10	30	38
Age – median (range)	42.5 (29 - 72)	43.5 (21 - 64)	41.5 (21 - 64)
Female – no. (%)	3 (30)	18 (60)	22 (58)
Days PCR to sample collection – median (range)	41 (16 - 45)	45 (32 - 56)	45 (32 - 56)
COVID-19 severity* – no. (%)			
Asymptomatic	3 (30)	7 (23)	13 (34)
Symptomatic outpatient	7 (70)	23 (77)	25 (66)
Positive antibody titer – no. (%)	9 (100)†	30 (100)	38 (100)
Antibody titer – median (range)	3.1 (1.8 - 9.8)†	5.4 (1.2 - 11.3)	4.8 (1.1 - 11.3)
**Characteristics**	**HCs without humoral immune response after infection**		
	**CoVac-1**	**SARS-CoV-2-specific EC**	**Cross-reactive EC**
Samples – no.	5	5	9
Age – median (range)	41 (34 - 55)	58 (33 - 66)	49 (33 - 66)
Female – no. (%)	4 (80)	4 (80)	7 (78)
Days PCR to sample collection – median (range)	43 (39 - 48)	44 (41 - 52)	44 (32 - 52)
COVID-19 severity* – no. (%)			
Asymptomatic	3 (60)	3 (60)	7 (78)
Symptomatic outpatient	2 (40)	2 (40)	2 (22)
Positive antibody titer – no. (%)	0 (0)	0 (0)	0 (0)
Antibody titer – median (range)	0.7 (0.3 – 1)	0.5 (0.5 – 1)	0.5 (0.2 – 1)

Abbreviations: HCs, healthy COVID-19 convalescents; PCR, polymerase chain reaction; EC, epitope composition; *COVID-19 severity categories 1) Asymptomatic: samples collected from individuals without symptomatic disease; 2) Symptomatic outpatient: sample collected from individuals reporting fever (≥ 38.0°C) and reported disease symptoms. SARS-CoV-2 IgG antibody titers were measured with a SARS-CoV-2 IgG ELISA (EUROIMMUN, 2606A_A_DE_C03, as constituted on 22 April 2020) assay (positivity was determined as a ratio ≥ 1.1)[2]. no., number. missing values: †n = 1.

Supplementary Table 7: Additional information on patients` prior vaccination.

	All	Phase I	Phase II
All participants – no. (%)	54 (100)	14 (100)	40 (100)
**Prev. application of approved vaccines – no. (%)**			
Vaccinated	45 (83)	9 (64)	36 (90)
mRNA-based	30 (67)	6 (67)	24 (67)
Adenoviral vector-based	5 (11)	2 (22)	3 (8)
Heterologous	10 (22)	1 (11)	9 (25)
Not vaccinated	8 (15)	5 (36)	3 (8)
n.a.	1 (2)	-	1 (2)
Number of vaccinations – median (range)	2 (1 - 4)	2 (2 - 4)	2 (1 - 4)
If vaccinated, no. of vaccinations prior to CoVac-1 – no. (%)			
One	2 (4)	-	2 (5)
Two	26 (58)	8 (89)	18 (50)
Three	15 (33)		15 (42)
Four	2 (4)	1 (11)	1 (3)
Time last COVID19-vaccination to CoVac-1 administration – median (range)	66 (28 - 234)	55 (28 - 129)	102 (29 - 234)

The messenger ribonucleic acid (mRNA)-based vaccine group includes samples from donors vaccinated either with mRNA-1273 or with BNT162b2. Donors from the adenoviral vector-based vaccine group received AZD1222 or Ad26.COV2.S. Donors of the heterologous vaccination regime group received one dose of AZD1222 or Ad26.COV2.S followed by at least one dose of mRNA-1273 or BNT162b2. no., number; n.a., not available.

Supplementary Table 8: Hematological malignancies and therapies of patients included in the study.

Diagnosis			N (%)
**All participants**			54 (100)
**FL**			11 (20)
	Lines of therapies	0	0 (0)
		1	1 (9)
		2 - 3	9 (82)
		> 3	1 (9)
	Types of therapy	Chemotherapy	0 (0)
		Chemo-immunotherapy	10 (91)
		Immunotherapy*	11 (100)
		Others†	2 (18)
	Anti-CD20 therapy	Discontinued within 6 months before vaccination	1 (9)
		Ongoing during vaccination	8 (73)
**CLL**			16 (30)
	Lines of therapies	0	4 (25)
		1	4 (25)
		2 - 3	7 (44)
		> 3	1 (6)
	Types of therapy	Chemotherapy	3 (19)
		Chemo-immunotherapy	5 (31)
		Immunotherapy	8 (50)
		Others†	12 (75)
	Anti-CD20 therapy	Discontinued within 6months before vaccination	1 (6)
		Ongoing during vaccination	0 (0)
**MCL**			13 (24)
	Lines of therapies	0	0 (0)
		1	0 (0)
		2 - 3	12 (92)
		> 3	1 (8)
	Types of therapy	Chemotherapy	0 (0)
		Chemo-immunotherapy	13 (100)
		Immunotherapy*	13 (100)
		Others†	10 (77)
	Anti-CD20 therapy	Discontinued within 6 months before vaccination	1 (8)
		Ongoing during vaccination	11 (85)
**DLBCL**			5 (9)
	Lines of therapies	0	0 (0)
		1	4 (80)
		2 - 3	0 (0)
		> 3	1(20)
	Types of therapy	Chemotherapy	0 (0)
		Chemo-immunotherapy	5 (100)
		Immunotherapy	0 (0)
		Others†	1 (20)
	Anti-CD20 therapy	Discontinued within 6 months before vaccination	1 (20)
		Ongoing during vaccination	1 (20)
**Other hematologic malignancies¥**			5 (9)
	Lines of therapies	0	0 (0)
		1	2 (40)
		2 - 3	3 (60)
		> 3	0 (0)
	Types of therapy	Chemotherapy	1 (20)
		Chemo-immunotherapy	2 (40)
		Immunotherapy*	2 (40)
		Others†	3 (60)
	Anti-CD20 therapy	Discontinued within 6 months before vaccination	0 (0)
		Ongoing during vaccination	1 (20)

*Including anti-CD20 maintenance therapy or combination with small molecules. †Radiotherapy, Hydroxycarbamide, small molecules (idelalisib, ibrutinib, venetoclax), CAR T-cell therapy, Nivolumab, autologous or allogenic transplantation. ¥Hodgkin`s lymphoma, marginal cell lymphoma, myeloproliferative syndrome, Waldenström`s macroglobulinemia. Abbreviations: CLL, chronic lymphocytic leukemia; DLBCL, diffuse large B-cell lymphoma; FL, follicular lymphoma; MCL, mantle cell lymphoma; n, number.

Supplementary Table 9: Mutations of SARS-CoV-2 omicron variants.

Protein	Position	Mutation	B.1.1.529.1 Omicron, BA.1	B.1.1.529.1.1 Omicron, BA.1.1	B.1.1.529.2 Omicron, BA.2	B.1.1.529.3 Omicron, BA.3
	135	S135R			x	x
	842	T842I			x	
	856	K856R	x	x		
	1307	G1307S			x	x
	2083	2083del	x	x		
	2084	L2084I	x	x		
	2710	A2710T	x	x		
	2789	L2789F			x	
	3090	T3090I			x	x
ORF1	3201	L3201F			x	
	3255	T3255I	x	x	x	x
	3395	P3395H	x	x	x	x
	3657	A3657V				x
	3675	SGF3675-3677del	x	x	x	x
	3758	I189V	x	x		
	4715	P4715L	x	x	x	x
	5716	R5716C			x	
	5967	I596V	x	x	x	x
	6564	T112I			x	
	19	T19I			x	
	24	L24S			x	
	25	PPA25-27del			x	
	27	A27S			x	
	67	A67V	x	x		x
	69	HV69-70del	x	x		x
	95	T95I	x	x		x
	142	G142D	x	x	x	x
	143	VYY143-145del	x	x		x
	211	N211I	x	x		x
	212	L212del	x	x		x
	213	V213G			x	
	214	ins214EPE	x	x		
	339	G339D	x	x	x	x
	346	R346K		x		
	371	S371L	x	x		
	371	S371F			x	x
	373	S373P	x	x	x	x
	375	S375F	x	x	x	x
	376	T376A			x	
	405	D405N			x	x
ORF2 spi	408	R408S			x	
	417	K417N	x	x	x	x
	440	N440K	x	x	x	x
	446	G446S	x	x		x
	477	S477N	x	x	x	x
	478	T478K	x	x	x	x
	484	E484A	x	x	x	x
	493	Q493R	x	x	x	x
	496	G496S	x	x		
	498	Q498R	x	x	x	x
	501	N501Y	x	x	x	x
	505	Y505H	x	x	x	x
	547	T547K	x	x		
	614	D614G	x	x	x	x
	655	H655Y	x	x	x	x
	679	N679K	x	x	x	x
	681	P681H	x	x	x	x
	764	N764K	x	x	x	x
	796	D796Y	x	x	x	x
	856	N856K	x	x		
	954	Q954H	x	x	x	x
	969	N969K	x	x	x	x
	981	L981F	x	x		
ORF3a	223	T223I			x	x
ORF4 env	9	T9I	x	x	x	x
	3	D3G	x	x		
ORF5 mem	19	Q19E	x	x	x	x
	63	A63T	x	x	x	x
ORF6	61	D61L			x	
	13	P13L	x	x	x	x
	31	ERS31-33del	x	x	x	x
ORF9 nuc	203	R203K	x	x	x	x
	204	G204R	x	x	x	x
	413	S413R			x	x

List of variant-defining and -associated mutations described for the four SARS-CoV-2 omicron variants (B.1.1.529.1-Omicron BA.1, B.1.1.529.1.1-Omicron BA.1.1, B.1.1.529.2-Omicron BA.2, B.1.1.529.3-Omicron BA.3).[12-15] Abbreviations: del, deletion; ins, insertion; nuc, nucleocapsid; spi, spike; env, envelope; mem, membrane; ORF, open reading frame.

Supplementary Figures

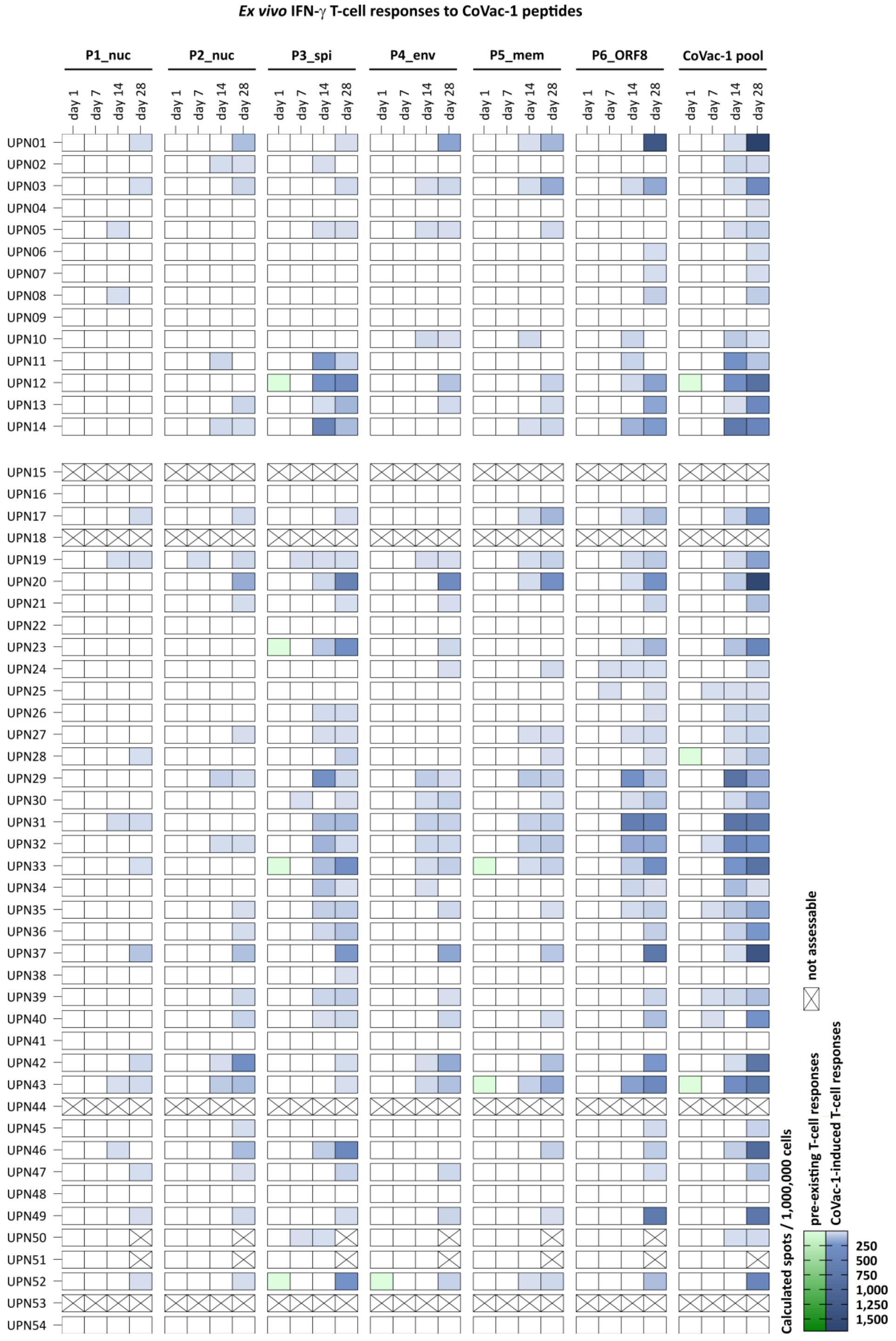

Supplementary Figure 1: Intensities of CoVac-1-induced T-cell responses *ex vivo* assessed in IFN-γ ELISPOT assays. Heatmap of pre-existing (color gradient green) or CoVac-1-induced (color gradient blue) T-cell response intensities to single CoVac-1 peptides (nuc, nucleocapsid; spi, spike; env, envelope; mem, membrane; ORF, open reading frame) and

CoVac-1 peptide pool in *ex vivo* IFN-γ ELISPOT assays using PBMCs from study patients (uniform patient number, UPN) before CoVac-1 application (day 1) and at different time points after vaccination (day 7, day 14, day 28). The first 14 study patients were included in the Phase I part of the trial. Ticked boxes display not assessable T-cell responses due to e.g. insufficient sample material or inability to mount antigen-specific T-cell responses (absence of CoVac-1-induced T-cell responses and no T-cell responses to HLA-DR T-cell epitope control panel including viral peptides from Epstein-Barr virus (EBV), cytomegalovirus (CMV) and adenovirus (ADV) assessed by IFN-γ ELISPOT assays).

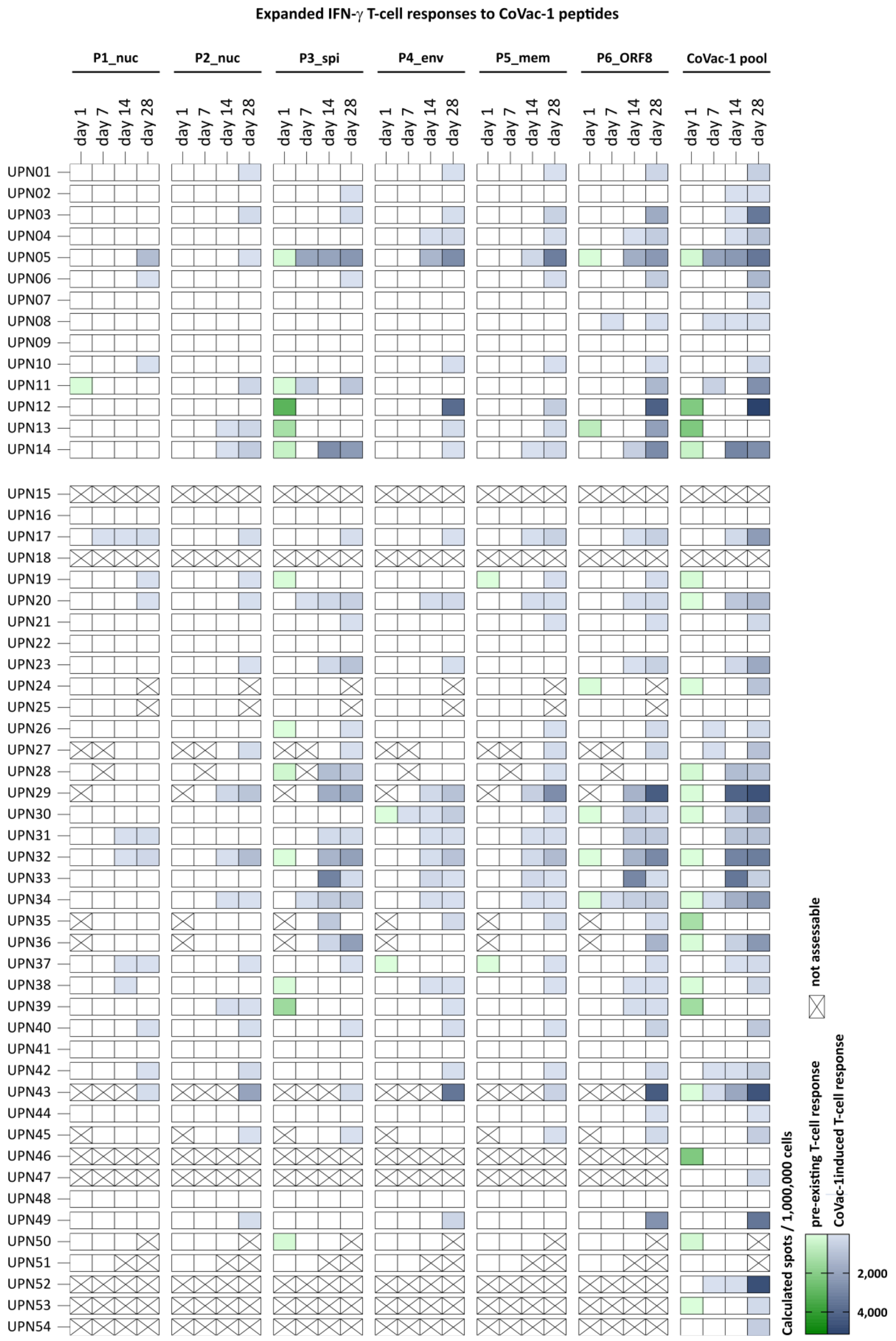

Supplementary Figure 2: Intensities of CoVac-1-induced T-cell responses assessed in IFN-γ ELISPOT assays after 12-day *in vitro* expansion. Heatmap of pre-existing (color gradient green) or CoVac-1-induced (color gradient blue) T-cell response intensities to single CoVac-1 peptides (nuc, nucleocapsid; spi, spike; env, envelope; mem, membrane; ORF, open reading frame) and CoVac-1 peptide pool in IFN-γ ELISPOT assays after 12-day *in vitro* expansion of PBMCs from study patients (uniform patient number, UPN) before (day 1) and at

different time points after CoVac-1 application (day 7, day 14, day 28). The first 14 study patients were included in the Phase I part of the trial. Ticked boxes display not assessable T-cell responses due to e.g. insufficient sample material or inability to mount antigen-specific T-cell responses (absence of CoVac-1 induced T-cell responses and no T-cell responses to HLA-DR T-cell epitope control panel including viral peptides from Epstein-Barr virus (EBV), cytomegalovirus (CMV) and adenovirus (ADV) assessed by IFN-γ ELISPOT assays).

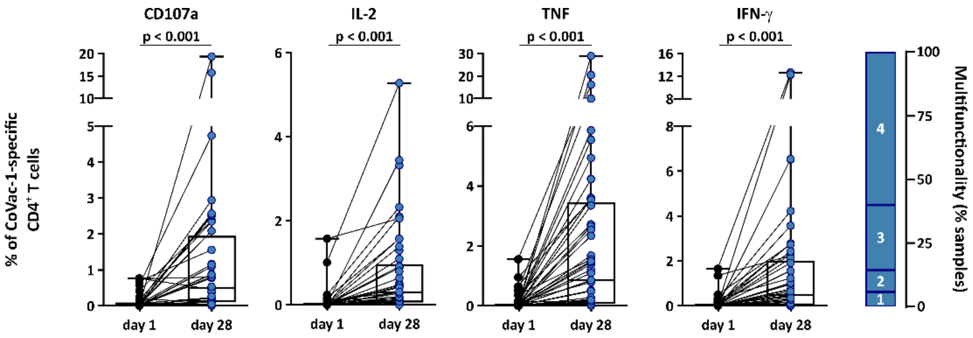

	CD107a day 1	CD107a day 28	IL-2 day 1	IL-2 day 28	TNF day 1	TNF day 28	IFN-γ day 1	IFN-γ day 28
No. (ind)	49	49 (22)	49	49 (31)	49	49 (32)	49	49 (34)
Median (pos)	0.01 (0.58)	0.38 (1.15)	0.0 (0.59)	0.28 (0.48)	0.01 (0.51)	0.86 (2.34)	0.0 (0.33)	0.48 (0.97)
% response		45		63		65		69

Supplementary Figure 3: CoVac-1-induced CD4+ T-cell responses in study participants. Frequencies of CoVac-1-specific CD4+ T cells in study participants (n = as indicated) prior to (day 1) or on day 28 following CoVac-1 administration after 12-day *in vitro* expansion. Functionality of CD4+ T cells was assessed for upregulation of the degranulation marker CD107a and production of the T-helper 1 (Th1) cytokines (IFN-γ, TNF, IL-2). The right graph displays the proportion of samples revealing bifunctional (2), trifunctional (3), or tetrafunctional (4) CD4+ T cells. Two-sided Wilcoxon signed-rank test. Combined box-line plots display median with 25th or 75th percentiles, and min/max whiskers. no, number; pos, positive; ind, induced.

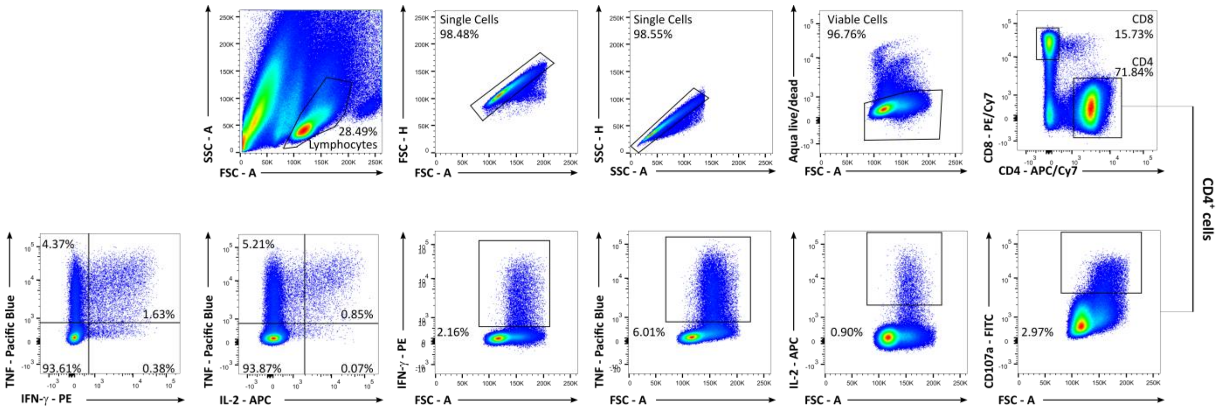

Supplementary Figure 4: Gating strategy for flow cytometry-based evaluation of surface marker and intracellular cytokine staining. Representative example showing the gating strategy for the evaluation of flow cytometry-acquired surface marker and intracellular cytokine staining data. The first gate identifies the lymphocytes (FSC-A vs. SSC-A), which are further gated for single cells (FSC-A vs. FSC-H and SSC-A vs. SSC-H) and viable cells (FSC-A vs. Aqua live/dead). $CD4^+$ T cells (CD4-APC/Cy7 vs. CD8-PE/Cy7) are analyzed for different cytokines (FSC-A vs. IFN-γ-PE; FSC-A vs. TNF-Pacific Blue; FSC-A vs. IL-2-APC; IFN-γ-PE vs. TNF-Pacific Blue; IL-2-APC vs. TNF-Pacific Blue) and the degranulation marker CD107a (FSC-A vs. CD107a-FITC). This gating strategy was applied for the data presented in Fig. 3f and Fig. S3.

Supplementary references

1 Bilich, T. *et al.* T cell and antibody kinetics delineate SARS-CoV-2 peptides mediating long-term immune responses in COVID-19 convalescent individuals. *Sci Transl Med* **13** (2021). https://doi.org:10.1126/scitranslmed.abf7517

2 Nelde, A. *et al.* SARS-CoV-2-derived peptides define heterologous and COVID-19-induced T cell recognition. *Nat Immunol* **22**, 74-85 (2021). https://doi.org:10.1038/s41590-020-00808-x

3 Heitmann, J. S. *et al.* A COVID-19 peptide vaccine for the induction of SARS-CoV-2 T cell immunity. *Nature* **601**, 617-622 (2022). https://doi.org:10.1038/s41586-021-04232-5

4 Bilich, T. *et al.* Preexisting and post-COVID-19 immune responses to SARS-CoV-2 in cancer patients. *Cancer Discov* (2021). https://doi.org:10.1158/2159-8290.CD-21-0191

5 Nilsson, J. B. *et al.* Machine learning reveals limited contribution of trans-only encoded variants to the HLA-DQ immunopeptidome. *Commun Biol* **6**, 442 (2023). https://doi.org:10.1038/s42003-023-04749-7

6 Rammensee, H. G. *et al.* Designing a SARS-CoV-2 T-Cell-Inducing Vaccine for High-Risk Patient Groups. *Vaccines (Basel)* **9** (2021). https://doi.org:10.3390/vaccines9050428

7 Rammensee, H. G. *et al.* A new synthetic toll-like receptor 1/2 ligand is an efficient adjuvant for peptide vaccination in a human volunteer. *J Immunother Cancer* **7** (2019). https://doi.org:10.1186/s40425-019-0796-5

8 Aucouturier, J., Dupuis, L., Deville, S., Ascarateil, S. & Ganne, V. Montanide ISA 720 and 51: a new generation of water in oil emulsions as adjuvants for human vaccines. *Expert Rev Vaccines* **1**, 111-118 (2002). https://doi.org:10.1586/14760584.1.1.111

9 Hilf, N. *et al.* Actively personalized vaccination trial for newly diagnosed glioblastoma. *Nature* **565**, 240-+ (2019). https://doi.org:10.1038/s41586-018-0810-y

10 Platten, M. *et al.* A vaccine targeting mutant IDH1 in newly diagnosed glioma. *Nature* (2021). https://doi.org:10.1038/s41586-021-03363-z

11 Tavares Da Silva, F. *et al.* Optimal approaches to data collection and analysis of potential immune mediated disorders in clinical trials of new vaccines. *Vaccine* **31**, 1870-1876 (2013). https://doi.org:10.1016/j.vaccine.2013.01.042

12 Yamasoba, D. *et al.* Virological characteristics of SARS-CoV-2 BA.2 variant. *bioRxiv*, 2022.2002.2014.480335 (2022). https://doi.org:10.1101/2022.02.14.480335

13 Kumar, S., Karuppanan, K. & Subramaniam, G. Omicron (BA.1) and Sub-Variants (BA.1, BA.2 and BA.3) of SARS-CoV-2 Spike Infectivity and Pathogenicity: A Comparative Sequence and Structural-based Computational Assessment. *bioRxiv*, 2022.2002.2011.480029 (2022). https://doi.org:10.1101/2022.02.11.480029

14 Abbas, Q., Kusakin, A., Sharrouf, K., Jyakhwo, S. & Komissarov, A. S. Follow-up investigation and detailed mutational characterization of the SARS-CoV-2 Omicron variant lineages (BA.1, BA.2, BA.3 and BA.1.1). *bioRxiv*, 2022.2002.2025.481941 (2022). https://doi.org:10.1101/2022.02.25.481941

15 Sun, Y., Lin, W., Dong, W. & Xu, J. Origin and evolutionary analysis of the SARS-CoV-2 Omicron variant. *J Biosaf Biosecur* **4**, 33-37 (2022). https://doi.org:10.1016/j.jobb.2021.12.001
