# Supplementary material for: Phase I/II trial of a peptide-based COVID-19 T-cell activator in patients with B-cell deficiency
Source: Nat Commun. 2023 Aug 18;14:5032. doi: 10.1038/s41467-023-40758-0 (PMC10439231; doi:10.1038/s41467-023-40758-0)
Supplement: Supplementary file 3 — Reporting Summary [file 41467_2023_40758_MOESM3_ESM.pdf]

Corresponding author(s): Juliane S. Walz

Last updated by author(s): Jul 28, 2023

Reporting Summary

Nature Portfolio wishes to improve the reproducibility of the work that we publish. This form provides structure for consistency and transparency in reporting. For further information on Nature Portfolio policies, see our Editorial Policies and the Editorial Policy Checklist.

Statistics

For all statistical analyses, confirm that the following items are present in the figure legend, table legend, main text, or Methods section.

n/a	Confirmed	
☐	☒	The exact sample size (n) for each experimental group/condition, given as a discrete number and unit of measurement
☐	☒	A statement on whether measurements were taken from distinct samples or whether the same sample was measured repeatedly
☐	☒	The statistical test(s) used AND whether they are one- or two-sided<br>*Only common tests should be described solely by name; describe more complex techniques in the Methods section.*
☒	☐	A description of all covariates tested
☒	☐	A description of any assumptions or corrections, such as tests of normality and adjustment for multiple comparisons
☐	☒	A full description of the statistical parameters including central tendency (e.g. means) or other basic estimates (e.g. regression coefficient) AND variation (e.g. standard deviation) or associated estimates of uncertainty (e.g. confidence intervals)
☐	☒	For null hypothesis testing, the test statistic (e.g. F, t, r) with confidence intervals, effect sizes, degrees of freedom and P value noted<br>*Give P values as exact values whenever suitable.*
☒	☐	For Bayesian analysis, information on the choice of priors and Markov chain Monte Carlo settings
☒	☐	For hierarchical and complex designs, identification of the appropriate level for tests and full reporting of outcomes
☒	☐	Estimates of effect sizes (e.g. Cohen's d, Pearson's r), indicating how they were calculated

Our web collection on statistics for biologists contains articles on many of the points above.

Software and code

Policy information about availability of computer code

Data collection	ImmunoSpot S6 analyzer, FACS Canto II cytometer, ADVIA Centaur XPT system
Data analysis	FlowJo10.7.1, GraphPad Prism 9.3.1, SAS 9.4, NetMHCIIpan 4.2a

For manuscripts utilizing custom algorithms or software that are central to the research but not yet described in published literature, software must be made available to editors and reviewers. We strongly encourage code deposition in a community repository (e.g. GitHub). See the Nature Portfolio guidelines for submitting code & software for further information.

Data

Policy information about availability of data

All manuscripts must include a data availability statement. This statement should provide the following information, where applicable:

- Accession codes, unique identifiers, or web links for publicly available datasets
- A description of any restrictions on data availability
- For clinical datasets or third party data, please ensure that the statement adheres to our policy

Data supporting the findings of this study including de-identified patient data are available after final completion of the trial report and are shared according to data sharing guidelines upon reasonable request to the corresponding author. Data supporting the findings of this study including de-identified patient data are available after final completion of the trial report and are shared according to data sharing guidelines upon reasonable request to the corresponding author J.S.W (juliane.walz@med.uni-tuebingen.de). Data will be only shared for non-commercial interests and after ethical approval. A data use agreement is obligatory.

Human research participants

Policy information about studies involving human research participants and Sex and Gender in Research.

Reporting on sex and gender	In total, 15 female and 39 male patients have been recruited. As the trial was not powered to provide subgroup analysis of each gender, this was not performed.
Population characteristics	Eligible participants were men or women aged 18 years or above, who signed the informed consent form. In addition, patients had primary or secondary antibody deficiency syndrome, defined by (i) IgG < 5.5 g/l, (ii) ongoing substitution of immunoglobulins for hypogammaglobulinemia, (iii) ongoing or up to 6 months after single agent anti-CD20 antibody therapy, (iv) ongoing or up to 6 months after combined anti-CD20 antibody therapy with Bruton`s tyrosine kinase (BTK)-inhibitors or B-cell lymphoma 2 (BCL2)-inhibitors, (v) after combined anti-CD20 antibody therapy with chemotherapy (e.g. fludarabine, cyclophosphamide, bendamustine, anthracycline, vincristine) (within 1-6 month post therapy) or (vi) anti-CD20 antibody maintenance therapy. All participants had to adhere to adequate contraception methods until three months after vaccination.<br>Exclusion criteria comprised: Pregnant or lactating females; participation in another clinical trial with a vaccine product; prior or current infection with SARS-CoV-2 (proven serologically or by PCR); persisting symptoms developed after vaccination against SARS-CoV-2 with one of the approved vaccines products; intention of receiving one dose of an already approved vaccine against SARS-CoV-2 before day 56; known previous anaphylactic reaction or hypersensitivity to any component of CoVac-1; patients presenting clinical, laboratory or radiological signs of tumor-progression; patients receiving active treatment with proteosome-inhibitors (e.g. bortezomib), or phosphoinositide-3-kinase-inhibors (e.g. idelalisib); relevant central nervous system (CNS) pathology or other neurological disease; positivity for human immunodeficieny virus (HIV) or active hepatitis; baseline CD4+ T-cell count ≤ 100/µL; chronic liver failure defined as Child-Pugh Score ≥ B; chronic renal failure (GFR < 40 mL/min/1.73 m2); serious cardiovascular disease (NYHA ≥ III); sickle cell anemia; or pre-existing auto-immune disease except for Hashimoto thyroiditis and mild psoriasis.<br>Only sex was considered for this trial, which was based on self-reported assessment.<br>In the trial patients aged 37-90 years, 28% male and 72% females, 8% with primary immunodeficiency and 92% with secondary immunodeficiency were included. Most patients with secondary immunodeficiency had CLL, followed by MCL and FL.
Recruitment	Participants were recruited at three study sites in Germany (the University Hospital Tübingen, Krankenhaus Nordwest, University Cancer Center, Frankfurt and Department of Hematology, Oncology and Cancer Immunology, Campus Benjamin Franklin, Charité-Universitätsmedizin Berlin). Patients were identified by local investigators during clinical routine or patients themselves contacted study sites. Once recruited participants were screened for eligibility. First Phase I of the trial was completed and after review of reactogenicity and immunogenicity by the data safety monitoring board and approval by the regulatory authorities (Paul Ehrlich Institute and local ethic committee), Phase II of the trial was initiated.
Ethics oversight	The trial was approved by the local ethics committees under the lead of the Ethics Committee at the University Hospital Tübingen (255/2021AMG1) and the competent authority Paul Ehrlich Institute prior to entrollement of any participant and performed in accordance with the International Council for Harmonization Good Clinical Practice guidelines. A second approval was obtained prior to recruiting in Phase II of the clinical trial. Prior to enrollment, all patients provided written informed consent.

Note that full information on the approval of the study protocol must also be provided in the manuscript.

Field-specific reporting

Please select the one below that is the best fit for your research. If you are not sure, read the appropriate sections before making your selection.

☒ Life sciences ☐ Behavioural & social sciences ☐ Ecological, evolutionary & environmental sciences

For a reference copy of the document with all sections, see nature.com/documents/nr-reporting-summary-flat.pdf

Life sciences study design

All studies must disclose on these points even when the disclosure is negative.

Sample size	The total sample size calculation (n = 54 patients) of the trial was based on the following assumptions: For the analysis of safety in the first 14 patients (Phase I), incidence of SAE associated with administration of CoVac-1 exceeding a predetermined rate of 20% was investigated. The trial was expanded to Phase II after proving safety and sufficient T-cell response (> 80% of patients, with documented CoVac-1-induced T-cell responses) measured by IFN-γ ELISPOT on day 28. Here, the sample size (n = 40) based on the assumption that, in the unfavorable case of SARS-CoV-2 specific immune response induction in ≤ 50% of the patients, the treatment concept is extended with a probability of at most 5%. On the other hand, in the favorable case of peptide-specific immune response induction in ≥ 70% of patients, the concept would be followed with a probability of at least 80%.
Data exclusions	Safety and immunogenicity data were available until day 56 and day 28 after vaccination, respectively. One patient missed the day 28 visit, and four patients were not assessable for immunogenicity data.

Replication	This is a report of an ongoing clinical trial. So far no attempt to replicate was performed.
Randomization	As this is single-armed clinical trial, no randomization was performed in this clinical trial.
Blinding	As this is single-armed clinical trial, there was no blinding in this clinical trial.

Reporting for specific materials, systems and methods

We require information from authors about some types of materials, experimental systems and methods used in many studies. Here, indicate whether each material, system or method listed is relevant to your study. If you are not sure if a list item applies to your research, read the appropriate section before selecting a response.

Materials & experimental systems

n/a	Involved in the study
☐	☒ Antibodies
☒	☐ Eukaryotic cell lines
☒	☐ Palaeontology and archaeology
☒	☐ Animals and other organisms
☐	☒ Clinical data
☒	☐ Dual use research of concern

Methods

n/a	Involved in the study
☒	☐ ChIP-seq
☐	☒ Flow cytometry
☒	☐ MRI-based neuroimaging

Antibodies

Antibodies used	APC/Cy7 anti-human CD4 (BioLegend, Cat# 300518, RRID: AB_314086), PE/Cy7 anti-human CD8 (Beckman Coulter, Cat# 737661, RRID: AB_1575980), Pacific Blue anti-human TNF (BioLegend, Cat# 502920, RRID: AB_528965), FITC anti-human CD107a, (BioLegend, Cat# 328606, RRID: AB_1186036), APC anti-human IL-2 (BioLegend, Cat# 500309, RRID: AB_315096), PE anti-human IFN-γ (BioLegend, Cat# 506507, RRID: AB_315440), anti-IFNγ antibody (clone 1-D1K, MabTech), anti-IFNγ biotinylated detection antibody (clone 7-B6-1, MabTech, Cat# 3420-6-250, RRID: AB_907273).
Validation	All antibodies were purchased from the above stated companies. Antibodies are well described and published elsewhere. Information can be sought from the manufactures website under the respective catalogue number.

Clinical data

Policy information about clinical studies

All manuscripts should comply with the ICMJEguidelines for publication of clinical research and a completedCONSORT checklist must be included with all submissions.

Clinical trial registration	The trial is registered under the ClinicalTrials.gov identifier NCT04954469.
Study protocol	The study protocol is provided with the submission of the manuscript.
Data collection	Data were collected at screening (up to 7 days before vaccination), day 1 (vaccination, baseline), day 7, day 14, day 28, and day 56. Both reactogenicity and immunogenicity were collected at indicated time points. In addition, participants reported on reactogenicity until day 28 by paper-based diary.<br>From July 6th, 2021 to January 13th, 2022, a total of 94 patients with congenital or acquired B-cell deficiency underwent screening. Data were collected from July 6th, 2021 to May 4th, 2022.
Outcomes	In this report, the primary endpoint immunogenicity is reported by the induction of CoVac-1-specific T-cell responses at day 28 assessed by IFN-γ ELISPOT assay.<br>Safety as endpoint is presented until day 56. Safety outcomes reflect the nature, frequency, and severity of solicited adverse events (AEs) until day 56 after vaccination. In addition, the number and percentage of participants with unsolicited events until day 56 were reported.<br>For Phase I, safety is the primary endpoint. For phase II, immunogenicity is primary endpoint and safety is secondary endpoint. Primary efficacy/immunogenicity endpoint was analysed for both phases together.<br>Furthermore, explorative endpoints such as characteristics of T-cell responses were analyzed.

Flow Cytometry

Plots

Confirm that:

- [x] The axis labels state the marker and fluorochrome used (e.g. CD4-FITC).
- [x] The axis scales are clearly visible. Include numbers along axes only for bottom left plot of group (a 'group' is an analysis of identical markers).
- [x] All plots are contour plots with outliers or pseudocolor plots.
- [x] A numerical value for number of cells or percentage (with statistics) is provided.

Methodology

Sample preparation	PBMCs were incubated with 10 µg/mL of peptide, 10 µg/mL Brefeldin A (Sigma-Aldrich), and a 1:500 dilution of GolgiStop (BD) for 12 - 16 h. Staining was performed using Cytofix/Cytoperm solution (BD), APC/Cy7 anti-human CD4 (BioLegend), PE/Cy7 anti-human CD8 (Beckman Coulter), Pacific Blue anti-human TNF, FITC anti-human CD107a, APC anti-human IL-2, and PE anti-human IFN-γ monoclonal antibodies (BioLegend). PMA (5 µg/mL) and ionomycin (1 µM, Sigma-Aldrich) served as positive control. Viable cells were determined using Aqua live/dead (Invitrogen).
Instrument	FACS Canto II cytometer (BD)
Software	FlowJo software version 10.7.1 (BD)
Cell population abundance	Not applicable.
Gating strategy	Viable cells were determined using Aqua live/dead (Invitrogen).

- [x] Tick this box to confirm that a figure exemplifying the gating strategy is provided in the Supplementary Information.
